# Characterization of Seminiferous Epithelium Stages in the Wild Javan Muntjac (*Muntiacus muntjak muntjak*) Using the Tubular Morphology Method

**DOI:** 10.1155/2018/3024532

**Published:** 2018-06-20

**Authors:** Sri Wahyuni, Gholib Gholib, I Ketut Mudite Adnyane, Muhammad Agil, Hamny Hamny, Srihadi Agungpriyono, Tuty Laswardi Yusuf

**Affiliations:** ^1^Laboratory of Anatomy, Faculty of Veterinary Medicine, Syiah Kuala University, Banda Aceh 23111, Indonesia; ^2^Laboratory of Physiology, Faculty of Veterinary Medicine, Syiah Kuala University, Banda Aceh 23111, Indonesia; ^3^Department of Anatomy, Physiology, and Pharmacology, Faculty of Veterinary Medicine, Bogor Agricultural University, Bogor 16680, Indonesia; ^4^Department of Clinic, Reproduction, and Pathology, Faculty of Veterinary Medicine, Bogor Agricultural University, Bogor 16680, Indonesia

## Abstract

Stages of the seminiferous epithelium of the testis of the wild Javan muntjac (*Muntiacus muntjak muntjak*) in hard antler period were characterized based on the tubular morphology method. The number and the relative frequencies of seminiferous epithelium stages and the morphometry of germinal cell nuclei were identified microscopically. We identified eight stages of seminiferous epithelium in testicular tissue of the Javan muntjac and found that the relative frequencies of stages I to VIII were 14.87, 15.12, 17.75, 6.87, 7.37, 12.37, 13, and 12.62%, respectively. The diameter of the nuclei of germinal cells varied in each stage of seminiferous epithelium. Diplotene-stage primary spermatocytes had prominent and large nuclei ~8.97 ± 1.0 *μ*m in stages III and IV. Pachytene primary spermatocytes appeared in most stages, except stage IV, whereas leptotene- and diplotene-stage primary spermatocytes were found in stages I and II, and III and IV, respectively. Round spermatids were observed in stages IV to VIII and in stage I but were absent in stages II and III, while elongated spermatids were observed in all stages except stage I. Our findings show that the stages of seminiferous epithelium in the Javan muntjac are similar to those found in neotropical cervids, small ruminants, and other domestic animals.

## 1. Introduction

The Javan muntjac (*Muntiacus muntjak muntjak*, Zimmermann 1780) is a small cervid belonging to the Cervidae family found in the Indonesian islands of Java and southern Sumatera. In addition to the Javan muntjac, there are five subspecies of muntjacs distributed in several regions and islands in Indonesia, such as* M. m. montanus* in Sumatra,* M. m. robinsoni *in the Riau Archipelago and Linga,* M. m. bancanus* in the Bangka and Belitung Islands,* M. m. pleicharicus* in the islands of Borneo, Bawal, Matasiri, and Java, and* M. m. nainggolani* in Bali and Lombok islands [1]. Although the IUCN conservation status of Indian muntjacs is low risk [2], all of these subspecies have been protected by the Indonesian Government since 1999 [1].

Study on the reproductive biology, particularly basic information associated with spermatogenesis in neotropical cervids, remains scarce [3], and it has not been reported in the Javan muntjac. The only reproductive study conducted so far on this subspecies evaluated sperm quality and spermatogenic activity during the velvet and hard antler periods using testicular biopsy techniques [4]. However, in that previous study, the stages of seminiferous epithelium could not be properly determined because the testicular samples obtained using a biopsy needle were very small. It is important to have a clear understanding of the spermatogenesis process as it provides an indispensable basis to monitor male fertility, which can be used to prevent a species from extinction through enhancing the reproductive capacity of males in various breeding programs.

Spermatogenesis is a long and complex process to produce male germ cells called spermatozoa. There are three phases of spermatogenesis, namely, the proliferative or spermatogonial phase, the meiotic or spermatocytary phase, and the differentiation or spermiogenic phase [5]. Spermatogenesis occurs in seminiferous tubules of testis, a unique site that contains three types of cells: male germ cells, Sertoli cells, and myoid cells [6]. Determination of seminiferous epithelium stages in male species is important not only to obtain quantitative information regarding spermatogenesis, but also to understand the normal process of spermatogenesis. For these purposes, there are two methods to characterize stages of the seminiferous epithelium cycle: (1) based on tubular morphology and (2) based on development of the acrosomic system and nuclear morphology of developing spermatids.

Using the tubular morphology method, eight stages of seminiferous epithelium have been reported in ruminants and other mammals, such as red deer* (Cervus elaphus) *[7], goat* (Capra hircus) *[8], donkey* (Equus asinus) *and mule* (Equus mulus mulus)* [9], landrace boar [10], roe deer* (Capreolus capreolus) *[11], wild boar [12], and ‘crioulo' horse [13]. In addition to the tubular morphology method, characterization of the stages of the seminiferous epithelium cycle can be performed by an acrosomic system using the periodic acid Schiff (PAS) staining. This method can better determine the size and position of spermatid acrosomes. Using this latter approach, characterization of the seminiferous epithelium stages is determined based on the presence, angle, and size of the acrosome, shape and location of spermatid nuclei, presence of meiotic division, and overall components of the testicular seminiferous epithelium. This method has been successfully applied in gerbil* (Meriones unguiculatus) *[14] and cynomolgus monkey (*Macaca fascicularis*) [15].

In the present study, we characterized the stages of the seminiferous epithelium cycle in the adult wild Javan muntjac. Our specific objectives were (1) to identify the stages of seminiferous epithelium, (2) to determine the relative frequency of each stage, and (3) to calculate nuclei diameters of germinal cells during the hard antler period in Javan muntjac.

## 2. Materials and Methods

### 2.1. Animals and Ethics Approval

This study used adult male Javan muntjacs captured from their natural habitat in Central Java Province, Indonesia, and then transported to Bogor Agricultural University, West Java Province, Indonesia. Our use of these Javan muntjacs for this study was under permission from the Ministry of Environment and Forestry, Republic of Indonesia (number: 23/Menhut-II/2011). An adult male was died the day after transportation, whereas another male was died due to fighting with other males during breeding program at Faculty of Veterinary Medicine, Bogor Agricultural University. Testicular samples of these males were then collected immediately and placed into Bouin's solution prior to tissue preparation for histological observations. Both Javan muntjacs were in hard antler period. The existence of hard antler growth from pedicles, which are a permanent extension of the frontal bone in cervid males, indicates that the animal has passed puberty [16].

### 2.2. Tissue Preparation

Testicular organs of two Javan muntjacs were collected, subsequently fixed using an immersion fixation technique in Bouin's solution for 24 h, and then transferred to 70% ethanol as a stopping point. Prior to histological tissue preparation, testes were dissected into small pieces (0.5 × 0.5 × 0.5 cm). Testicular samples were then dehydrated in a graded ethanol series of 70%, 80%, 90%, and absolute, cleared in xylene, and immersed in paraffin infiltration, followed by embedding in a paraffin block. Paraffinized tissue was cut into 4-*μ*m-thick sections using a Leica RM2235 manual microtome (Leica Biosystems, Nussloch GmbH, Germany) and placed on clean glass slides. Afterwards, paraffin was removed using a xylene solution, rehydrated, and subsequently stained with hematoxylin and eosin (H&E). All sections were dehydrated, cleared, and mounted with cover slips and Entellan® (Merck, Germany).

### 2.3. Stages of Seminiferous Epithelium and Determination of Relative Frequency

The stages of seminiferous epithelium identified in this study were based on the tubular morphology method that focuses on the shape and position of spermatid nuclei, meiotic division of spermatocytes, and the presence of other seminiferous epithelium components [17, 18]. Each stage was photographed using a BX41 light microscope (Olympus, Japan) equipped with a DP12 digital camera (Olympus, Japan). Furthermore, the relative frequency of each stage was determined using 800 cross-sectional seminiferous tubules (400 cross sections per animal) referring to the staging of Almeida et al. [12]. Seminiferous tubules were observed using a CH30 light microscope (Olympus, Japan). Data are presented as the mean ± standard deviation (SD) and percentages.

### 2.4. Nuclei Measurements of Germinal Cells

The diameters of germinal cell nuclei were calculated by measuring 20 nuclei of each cell type at cross sections of seminiferous tubule stages, with the exception of elongated spermatids, in which the lengths of nuclei in the cells were measured. Additionally, nuclear diameter of type A spermatogonium which has a flattened nucleus was measured by two different direction, i.e., vertical and horizontal, and then calculated as average of diameter of type A spermatogonium. In total, 20 cells of type A spermatogonium for each of the observed stages were measured. Measurements of nuclei diameter and length of cells were performed using ImageJ 1.34 and data was presented as mean ± SD.

## 3. Results

### 3.1. Stages of Seminiferous Epithelium Cycle

**Figure 1:**
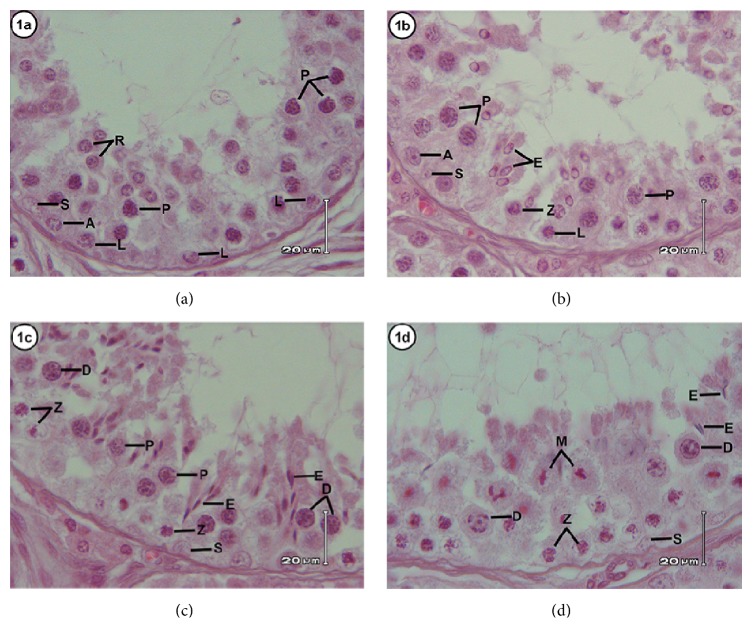
Stages I to IV of the cycle of the seminiferous epithelium according to the tubular morphology method.** Stage I (a)** shows type A spermatogonia (A), leptotene spermatocytes (L), pachytene primary spermatocytes (P), round spermatids (R), and Sertoli cells (S).** Stage II (b)** consists of type A spermatogonia (A), leptotene spermatocyte (L), zygotene spermatocyte (Z), pachytene spermatocytes (P), elongating spermatids (E), and Sertoli cells (S).** Stage III (c)** presents zygotene spermatocytes (Z), pachytene spermatocytes (P), diplotene spermatocytes (D), elongated spermatids (E), and Sertoli cells (S).** Stage IV (d)** shows zygotene spermatocytes (Z), diplotene spermatocytes (D), elongated spermatids (E), meiosis division (M), and Sertoli cells (S). HE staining with scale bar: 20 *μ*m.

**Figure 2:**
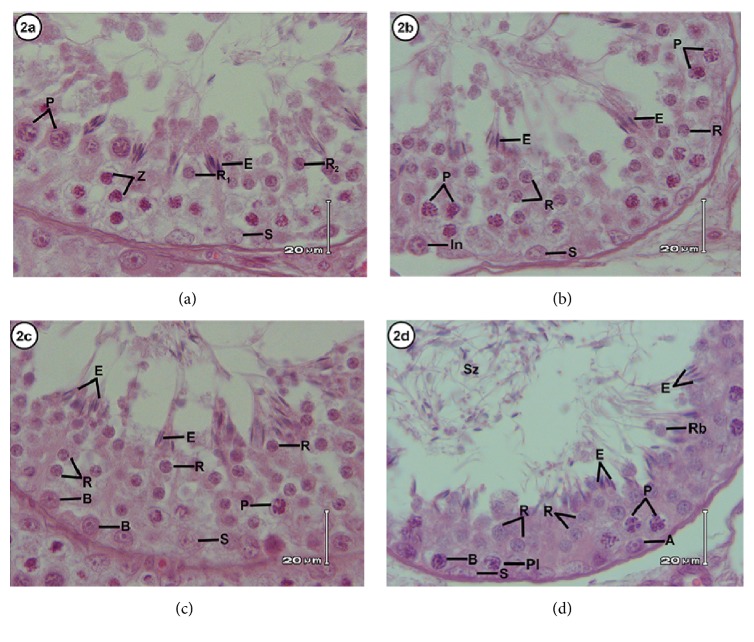
Stages V to VIII of the seminiferous epithelium based on the tubular morphology method.** Stage V (a)** shows zygotene spermatocytes (Z), pachytene spermatocytes (P), round spermatids generation 1 (R_1_) and generation 2 (R_2_), elongated spermatids (E), and Sertoli cells (S).** Stage VI (b)** presents intermediate spermatogonia (In), pachytene spermatocytes (P), round spermatids (R), elongated spermatids (E), and Sertoli cells (S).** Stage VII (c) **shows type B spermatogonia (B), pachytene spermatocytes (P), round spermatids (R), elongated spermatids (E), and Sertoli cells (S).** Stage VIII (d) shows **type A spermatogonia (A) and B spermatogonia (B), preleptotene (Pl), pachytene (P), round spermatids (R), elongated spermatids (E), Sertoli cells (S), spermatozoa (Sz), and residual bodies (Rb). HE staining with scale bar: 20 *μ*m.

The cycle of seminiferous epithelium in the Javan muntjac can be divided into eight stages according to the tubular morphology approach (Figures 1 and 2). Characterization of each stage is described as follows.

#### 3.1.1. Stage I (Figure 1(a))

This stage is characterized by the absence of elongated spermatids. The prominent cell was an early generation of spermatids with round nuclei found in the upper layer of seminiferous epithelium. Type A spermatogonia at the basal lamina of seminiferous tubules, as well as primary spermatocytes in the leptotene and pachytene stages of prophase I, were also observed in the seminiferous epithelium. Additionally, Sertoli cells were found among germinal cells with ovoid and pale nuclei and also conspicuous nucleoli.

#### 3.1.2. Stage II (Figure 1(b))

The main characteristic of this stage was the presence of spermatids with elongating nuclei, leading to nuclei of Sertoli cells. Type A spermatogonia with a similar morphology to those found in the previous stage were also observed at this stage. Leptotene-stage primary spermatocytes were located in the area adjacent to the basal lamina. The other germinal cells in this stage were primary spermatocytes in the zygotene and pachytene stage, which were clearly observed in the seminiferous epithelium.

#### 3.1.3. Stage III (Figure 1(c))

A number of elongated spermatids in groups were found adjacent to the basal lamina and directly towards nuclei of Sertoli cells. The presence of primary spermatocytes at two different stages, specifically zygotene and diplotene, with large nuclei observed in spermatocytes in the diplotene stage, was the other notable characteristic of this stage. Type A spermatogonia were also observed in the seminiferous epithelium.

#### 3.1.4. Stage IV (Figure 1(d))

This stage was characterized by the presence of meiotic division of diplotene-stage primary spermatocytes to secondary spermatocytes subsequently divided to produce round spermatids. At the basal lamina, type A spermatogonia were observed, while elongated spermatids and primary spermatocytes in the zygotene stage were detected at the adluminal region of the seminiferous epithelium.

#### 3.1.5. Stage V (Figure 2(a))

Elongated spermatids were found in groups located in the crypts of Sertoli cells. Two generations of round spermatids as well as zygotene and pachytene stages of primary spermatocytes were also observed in this stage. Type A spermatogonia and nuclei of Sertoli cells appeared at the basal lamina of the tubule.

#### 3.1.6. Stage VI (Figure 2(b))

The main characteristic of this stage was the position of elongated spermatids found closer to the tubule lumen. All types of germinal cells from the previous stage were found in this stage, except for zygotene-stage primary spermatocytes. The large nuclei of spermatocytes in the pachytene stage were located in the middle of the seminiferous epithelium, while the nuclei of Sertoli cells, type A spermatogonia, and intermediate spermatogonium were closer to the basal lamina of the seminiferous tubule.

#### 3.1.7. Stage VII (Figure 2(c))

In this stage, the elongated spermatids were closer to the tubular lumen and were dissociated from each other. Spermatocytes in the pachytene stage have larger nuclei compared to those found in stage VI. Other germinal cells were observed at this stage, namely, round spermatids, type A and type B spermatogonia, and crypts and Sertoli cell nuclei.

#### 3.1.8. Stage VIII (Figure 2(d))

The important characteristic of this stage was spermiation of the elongated spermatids into the lumen of seminiferous tubule as spermatozoa. Round spermatids, spermatocytes in preleptotene and pachytene stages, nuclei of Sertoli cells, and type A and type B spermatogonia were also observed in this stage.

### 3.2. Relative Frequency of Seminiferous Epithelium Stages

The relative frequency of each stage of the seminiferous epithelium cycle of the testis is presented in Table 1. The highest frequency (mean ± SD and percentages) was observed in stage III with 71 ± 5.7 (17.75%), followed by stage II with 60.5 ± 7.8 (15.12%), stage I with 59.5 ± 3.5 (14.87%), stage VII with 52 ± 2.8 (13%), stage VIII with 50.5 ± 3.5 (12.62%), stage VI with 49.5 ± 3.5 (12.37%), stage V with 29.5 ± 3.5 (7.37%), and stage IV (meiotic phase) with 27.5 ± 4.9 (12.37%). In a broad context, the premeiotic phase (stages I, II, and III), the meiotic phase (stage IV), and the postmeiotic phase (stages V, VI, VII, and VIII) account for 47.75%, 6.87%, and 45.37% of the seminiferous epithelium cycle, respectively.

### 3.3. Nuclei Diameters of Germinal Cells

Diameters of germinal cell nuclei (mean ± SD) at each stage are presented in Table 2. We found that the type A spermatogonia have relatively similar diameters at all stages with a range of 4.19 ± 0.12 *μ*m in stage I to 4.64 ± 0.44 *μ*m in stage VII. The average diameter of preleptotene-stage primary spermatocytes was 5.15 ± 0.66 *μ*m, which were only observed in stage VIII. Furthermore, the most abundant primary spermatocyte, which was present in almost all stages, was the pachytene stage with a diameter range of 6.39 ± 0.35 *μ*m in stage VI to 7.81 ± 0.36 *μ*m in stage II. However, this cell was absent in stages IV, while primary spermatocytes at the diplotene and leptotene stages were only found in stages III and IV, and in stages I and II, respectively. Furthermore, we found that round spermatids were present in stages IV to VIII and in stage I but absent in stages II and III, whereas elongated spermatids appeared in stage II and then distributed in stages III to VIII, but not found in stage I.

## 4. Discussion

The present study was the first to investigate and characterize stages of the seminiferous epithelium and its relative frequency in the wild Javan muntjac. The data obtained from this study provide valuable information for further studies focusing on or related to breeding programs of Javan muntjac in captivity to protect the species. Based on our observations using the tubular morphology method, we identified and characterized eight distinct stages of seminiferous epithelium in this study. These findings are consistent with those described for many mammalian species reported previously, such as the brown brocket deer [3], red deer [7], landrace boar [10], roe deer [11], wild boar [12], and rusa deer* (Cervus timorensis)* [19]. In contrast to primates, for example, marmosets, two or more cellular associations (stages of seminiferous epithelium) were identified from a cross section of the seminiferous tubule [18], because spermatogenesis as in marmoset is asymmetrically distributed, resulting in more than one stage per cross section of seminiferous tubule [20].

As shown in Table 1, our combined relative frequencies of seminiferous epithelium stages grouped into premeiotic (47.75%), meiotic (6.87%), and postmeiotic (45.37%) phases are similar to those found in goats (49.1%, 10.7%, and 40.2%, respectively) by França et al. [21]. In addition, our findings show that the seminiferous epithelium was more frequent in the premeiotic phase compared to the meiotic and postmeiotic phases, which is similar to results reported in other ruminants such as goats [8, 21] and swamp buffaloes [22]. In contrast, the postmeiotic phase of the seminiferous epithelium was reported to be more frequent than the premeiotic and meiotic phases in donkeys and mules [9] and also in llamas* (Lama glama) *[23]. In red deer, there are no major differences among the relative frequency of all stages of the seminiferous epithelium, although during breeding season it was reported that the premeiotic phase had the highest frequency [7]. In other temperate deer such as roe deer, the complete stage of the spermatogenic cycle was found in the rutting period (May–August), but in the postrutting period (November) the seminiferous epithelium was aligned only by Sertoli cells and spermatogonia [24]. These differences in the relative frequency of premeiotic, meiotic, and postmeiotic phase length in Javan muntjac and other ruminants may be related to the lifespan of primary spermatocytes, which is longer than that of other germinal cells [25]. Our determination of the relative frequency of seminiferous epithelium stages is an important indicator for assessing pathological conditions on kinetic changes of spermatogenesis [26], which subsequently influences daily sperm production [20].

Germinal cell association in each stage showed the pattern of spermatogonia development from stage I to stage VIII. This pattern is similar to the germinal cell association found in goats [8, 21] and rams [27]. Type A spermatogonia present from stages I to VI subsequently differentiated to type B spermatogonia in stages VII and VIII, which are characterized by a thicker nucleus and condensed heterochromatin. Furthermore, in stage VIII, type B spermatogonia developed into preleptotene stage of primary spermatocytes. Interestingly, this pattern of spermatogonia development in the Javan muntjac differs from that found in goats [21] in which, during stage VI, these cells were observed as intermediate spermatogonia before differentiating into type B spermatogonia in stages VII and VIII. However, the cellular composition of the testicular seminiferous tubule in the Javan muntjac is similar to that in rams [27] and landrace boar [10]. As a result of secondary spermatocyte division, first-round spermatids appeared in stages IV, then stages V to VIII, and stage I. Furthermore, the lifespan of a secondary spermatocyte is very short [28] (e.g., 6 h in human) [25] and, therefore, it is inherently difficult to observe this process. Additionally, the shape of round spermatids begins long, namely, elongating spermatids, in stage II and they subsequently differentiate into elongated spermatids. After their nucleus condenses and is covered by an acrosome cap, elongated spermatids differentiate into spermatozoa, followed by their release into the lumen of the seminiferous tubule.

We found that nuclei diameters of germinal cells varied in each stage of the seminiferous epithelium. In general, germinal cells nuclei in the Javan muntjac were smaller than those in beef bull [29]. For example, the diameters of pachytene-stage primary spermatocytes and spermatids in cattle were 11.4 ± 0.4 *μ*m and 7.5 ± 0.5 *μ*m, respectively. Measurements of germinal cell nuclei diameter in hard antler are important to understand sperm production potency during antler periods. In the future, a comparison between our measurements of germinal cell diameter in the Javan muntjac during the hard antler period and those in the velvet antler period is warranted to provide a better understanding of the potency of sperm production during both antler stages. For instance, in cattle, germinal cell diameter can be used to assess the effect of food supplementation on the degeneration rate of germinal cells, which relates to sperm production rate [30].

Although this study was specifically conducted using data from the hard antler period of the wild Javan muntjac, our findings provide complete data of all seminiferous epithelium stages (I to VIII). For seasonal cervids, gonadal morphology and histology, testosterone levels, and behavior are influenced by season. Similarly, in mammals, the process of spermatogenesis varies among species and also changes with the season. Furthermore, environmental temperature and hormone level fluctuations are two factors that influence spermatogenesis [31] as reported in red deer [32], fallow deer [33], and sika deer [34, 35]. When hard antler roe deer enter the rutting period in May, they exhibit elevated testosterone levels and increased sexual behavior, and spermatogenesis was complete [36]. Another study of roe deer by Roelants et al. [37] reported peak testosterone levels in August resulting in a maximum meiotic intensity of spermatogenesis, which is proof of increased sperm production. Similar results have also been reported in red deer. In contrast, sperm production decreases during the velvet antler period when testosterone levels decrease drastically [7]. In several species, however, male cervids are still fertile in the velvet antler as reported in chital deer [38], reeves muntjac* (M. m. reevesi) *[39], Formosan muntjac* (M. reevesi micrurus) *[40], and brown brocket bucks* (Mazama gouazoubira) *[3]. We propose that the Javan muntjac follows a similar reproductive pattern as those cervids in the context that the complete eight stages of seminiferous epithelium observed in the hard antler period are also found in the velvet antler period. This premise is supported by a previous study in Javan muntjac that reported that high concentrations of spermatozoa were found in ejaculates during both the hard and velvet antler periods [4]. However, future studies are needed to investigate whether there are differences in germinal cell association in each stage of spermatogenesis between the velvet and the hard antler of Javan muntjac.

## 5. Conclusion

The eight stages of the seminiferous epithelium and their relative frequency in the Javan muntjac in the hard antler period in the present study are similar to that of neotropical cervids ruminants and other domestic animals. Our findings provide valuable information on the basic reproductive biology of the Javan muntjac and may thereby support breeding programs for this species.

## Figures and Tables

**Table 1:** Frequency of eight stages (I-VIII) of seminiferous epithelium in the Javan muntjac during hard antler period.

Stages and phase of seminiferous epithelium	Mean±SD	Frequency (%)
Stage I	59.5±3.5	14.87
Stage II	60.5±7.8	15.12
Stage III	71.0 ±5.7	17.75
Stage IV	27.5±4.9	6.87
Stage V	29.5±3.5	7.37
Stage VI	49.5±3.5	12.37
Stage VII	52.0 ±2.8	13.0
Stage VIII	50.5±3.5	12.62
Premeiotic phase (stages I to III)	-	47.75
Meiotic phase (stage IV)	-	6.87
Postmeiotic phase (stages V to VIII)	-	45.37

**Table 2:** The diameter (mean ± SD) of germinal cell nuclei in eight stages (I-VIII) of seminiferous epithelium in the Javan muntjac during hard antler period.

Germinal cells	Diameter of nucleus (*μ*m)/stage							
	I	II	III	IV	V	VI	VII	VIII
Spermatogonia A	4.19 ± 0.12	4.29 ± 0.12	4.36 ± 0.43	4.42 ± 0.41	4.50 ± 0.12	4.57 ± 0.13	4.64 ± 0.44	4.59 ± 0.36
Primary spermatocyte								
(a) preleptotene	-	-	-	-	-	-	-	5.15 ± 0.66
(b) leptotene	5.29 ± 0.25	5.33 ± 0.52	-	-	-	-	-	-
(c) zygotene	-	5.62 ± 0.73	5.76 ± 0.42	5.98 ± 0.53	6.02 ± 0.89	-	-	-
(d) pachytene	7.18 ± 0.33	7.81 ± 0.36	7.76 ± 0.47	-	6.94 ± 0.41	6.39 ± 0.35	6.59 ± 0.29	6.92 ± 0.48
(e) diplotene	-	-	8.97 ± 1.01	8.72 ± 0.17	-	-	-	-
Round spermatid	5.32 ± 0.38	-	-	5.84 ± 0.12	5.63 ± 0.41	5.32 ± 0.32	5.81 ± 0.37	5.56 ± 0.14
Elongated spermatid^*∗*^		4.35 ± 0.19	5.19 ± 0.43	5.44 ± 0.37	6.05 ± 0.36	6.43 ± 0.38	5.78 ± 0.53	5.71 ± 0.35

^*∗*^Length of elongated spermatid nuclei.

## Data Availability

The data used to support the findings of this study are available from the corresponding author upon request.
